# Single-cell RNA sequencing depicts metabolic changes in children with aplastic anemia

**DOI:** 10.3389/fonc.2023.1075408

**Published:** 2023-03-29

**Authors:** Qin Zhou, Lifen Huang, Yong Liu, Junbin Huang, Luping Wen, Jing Yang, Jintang Liang, Yun Chen, Chun Chen

**Affiliations:** Pediatric Hematology Laboratory, Division of Hematology/Oncology, Department of Pediatrics, The Seventh Affiliated Hospital of Sun Yat-Sen University, Shenzhen, Guangdong, China

**Keywords:** aplastic anemia (AA), cell metabolism, scRNA seq, T lymphocytes, NK cells, NENF

## Abstract

**Introduction:**

Aplastic anemia (AA) is a bone marrow hematopoietic failure syndrome mediated by immune cells. The mechanism of this immune disorder is not well understood and therapeutic strategies still need to be improved.

**Methods:**

Studies have found that abnormalities in metabolisms promote the survival of AA cells. In recent years, an increasing number of studies have reported the immunosuppressive therapy for the treatment of AA. In this study, we analyzed the transcriptome of AA from peripheral blood compared with healthy donors by single-cell sequencing and identified the affected metabolic pathways including lysine degradation. We demonstrated that the metabolic abnormalities of T lymphocytes mainly focus on glycolysis/gluconeogenesis. In addition, the metabolic abnormalities of natural killer cells concentrated in oxidative phosphorylation.

**Results:**

The key genes involved in abnormal metabolic processes were Neustein neurotrophic factor (*NENF*), inositol polyphosphate-4-phosphatase type II B (*INPP4B*), aldo-keto reductase family 1, member C3 (*AKR1C3*), and carbohydrate (N-acetylglucosamine-6-O) sulfotransferase 2 (*CHST2*) by differential gene expression analysis.

**Discussion:**

Molecule interaction analysis showed that tumor necrosis factor superfamily, member 12 (TNFSM12) in tumor necrosis factor (TNF) signaling was broadly activated in AA. In conclusion, we suppose that the treatment of the immune cells’ abnormal metabolic pathway may contribute to the development of novel strategies to treat AA.

## Introduction

Aplastic anemia (AA) is a bone marrow hematopoietic failure syndrome caused by various etiologies. It is characterized by a decreased proliferation of bone marrow hematopoietic cells and peripheral blood pancytopenia (1). The main clinical manifestations are anemia, hemorrhage, and infection (2). The pathogenesis of AA is complicated, including the abnormality of the hematopoietic microenvironment (3), deficiency of hematopoietic stem cells/progenitor cells (4), and disorders of the immune system (5). AA occurs at any age. However, compared with adults, a large proportion of children with AA have a relatively high incidence rate of the bone marrow failure syndrome (6). The incidence of AA varies geographically, which is two-to-three times higher in Asia than in the western world (7).

Most acquired AA is considered to be the destruction of bone marrow hematopoietic cells mediated by T cells (8). Early studies showed that removing lymphocytes from the bone marrow with AA could increase the number of cell colonies in tissue culture, while adding the same lymphocytes to a normal bone marrow would inhibit *in vitro* hematopoiesis (9). In clinic, human leukocyte antigen (HLA)-matched sibling bone marrow transplantation is the first-line treatment for AA patients under 40 years old (10). The combined immunosuppressive therapy of eltrombopag, thymoglobulin, and cyclosporine A is the initial treatment of refractory AA, and patients’ survival rate is approximately 90% (11).

Cell metabolism promotes the absorption of nutrients and various components required for cell synthesis (12), enabling organisms to grow and reproduce, maintain their structure, and react to the external environment (13). In terms of obtaining energy and biosynthesis, aerobic glycolysis is very important for cell proliferation (14). The transformation of intracellular metabolic pathways in immune cells alters the function of immune cells (15). In immune cells, six metabolic pathways were intensively discussed: glycolysis, the tricarboxylic acid cycle, the pentose phosphate pathway, fatty acid oxidation, fatty acid synthesis, and amino acid metabolism (15). Amino acid metabolism plays an important role in regulating the function of innate and adaptive immune systems. Previous investigations indicate that the deletion of the transporter Alanine/Serine/Cysteine Transporter 2 (*ASCT2*) (responsible for glutamine and leucine uptake) gene in T cells will damage the function of helper T cell 1 (TH1) and helper T cell 17 (TH17) cells (16). The transition of the metabolic pathway from oxidative phosphorylation to aerobic glycolysis is a sign of T-cell activation and a key step to satisfy the metabolic requirements in the process of cell proliferation (17). Studies have shown that the glucose analog 2-deoxyglucose, an inhibitor of the glycolysis pathway, inhibits T cells from developing into TH17 cells (18). Therefore, metabolic reprogramming is critical for T-cell activation and functional execution (19). In order to promote drug uptake and enhance the delivery ability to target T-cell populations, we coupled them with glucose transporters (20). Therefore, regulating the metabolism of T cells may be a therapeutic means to treat AA.

Single-cell messenger RNA (mRNA) sequencing (scRNA-seq) is a technology for an unbiased, high-throughput, and high-resolution transcriptome analysis of cell heterogeneity in populations (21). It aggregates cells and identifies new subsets, as well as gene expression in various tissues (22). We created a thorough transcriptional map of immune cells from healthy controls and patients with AA by single-cell RNA sequencing. Then, we explored the changes of the cellular transcriptome in patients and identified the key metabolic pathways that may affect the occurrence of AA.

## Materials and methods

### Clinical samples

The transcriptomic profile was obtained from peripheral blood from five children with AA (AA: LJX-AA, LZL-AA, SLT-AA, WJL-AA, and XF-AA) and three healthy donors (Ctrl: CBC-Ctrl, CRC-Ctrl, and LJJ-Ctrl) (**Figure S1**). All samples were recruited *via* The Seventh Affiliated Hospital of Sun Yat-sen University. All participants provided written informed consent before inclusion in the study.

### Single-cell mRNA library preparation and sequencing

The complementary DNA (cDNA)/DNA/small RNA libraries were sequenced on the Illumina sequencing platform by Genedenovo Biotechnology Co., Ltd (Guangzhou, China). Cellranger was used to remove the reads with low sequencing quality and then compare them with the reference genome to annotate as a specific gene. After unique molecular identifiers (UMI) correction and statistics, the unfiltered feature barcode matrix was obtained. The cells in the data were identified and distinguished according to the unfiltered feature barcode array. To filtrate multicellular samples, Doublet-Finder was applied to calculate the gel beads in emulsion (GEM) probability [pattern analysis and neural networks (pANN) value]. In addition, we used the following indicators to perform cell filtration: the number of genes identified in a single cell (200.0–3,600.0), the total number of UMI in a single cell (<17,000.0), and the proportion of mitochondrial gene expression in a single cell (<25.0%).

### Data analysis and visualization

We conducted Harmony for data consolidation and batch effect correction. Dimension reduction, cell clustering, and differential gene expression were performed using the Seurat package. Based on the subset information of cells, we set | log2FC | ≥ 0.36 and the proportion of cells expressing target genes in each group ≥ 0.1 as the threshold. Then, we used the modeling and simulation team (MAST) obstacle model to test the significance of differences. We screened the pathway with the enrichment degree of top 20 in kyoto encylopaedia of genes and genomes (KEGG) A class Metabolism from the KEGG enrichment of clusters (Ctrl *vs*. AA). We calculated the number of differential genes in each term in the Gene Ontology (GO) database (http://www.geneontology.org/) and applied a hypergeometric test to find the GO entries that are significantly enriched. The Kyoto Encyclopedia of Genes and Genomes pathway was performed by Omicshare tools (http://www.omicshare.com/tools/). Gene Set Enrichment Analysis (GSEA) was performed by using the software GSEA and MSigDB (23). Disease Ontology Analysis was performed by the disease ontology (DO) database (http://disease-ontology.org/). The CellPhoneDB package was used to estimate cell–cell communication.

### Statistical analysis

Data visualizing and statistical analysis were performed using GraphPad Prism 8.0 (GraphPad Software Inc, CA, USA). Differences between experimental groups were analyzed using unpaired Student’s t-test. P value < 0.05 was considered significant.

## Results

### Single-cell analysis and cell type identification

**Figure 1:**
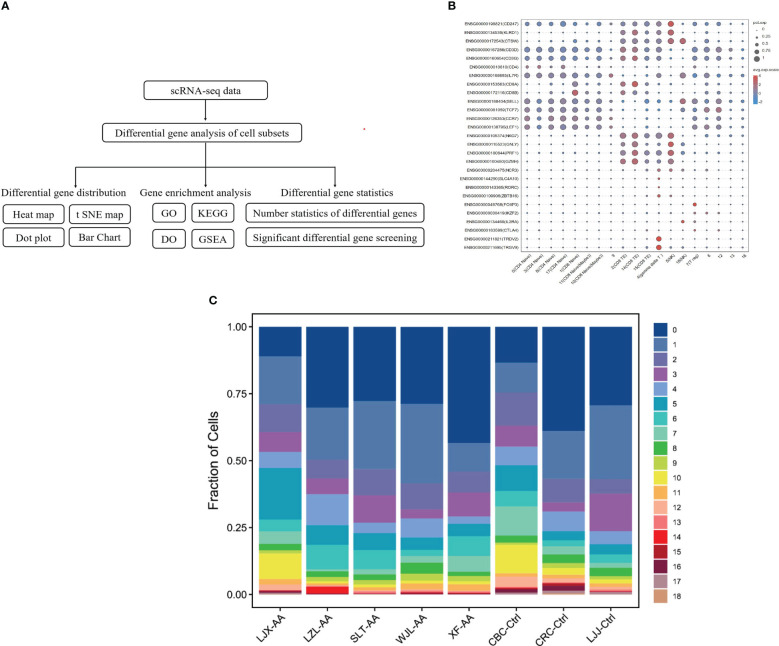
Identify cells and classify subsets with single-cell analysis. Schematic representation of the experimental design and single-cell mRNA sequencing (scRNA-seq) sequencing procedure **(A)**. The surface marker of immune cells was used to identify cells and classify subsets. Dot sizes represent percent expressed and dot colors represent the average-expressed scale **(B)**. Fraction of cell subsets’ differences between groups. Colors indicate cell clusters with numbered labels **(C)**.

To interrogate the metabolism of immune cells in patients with AA, clinical peripheral blood specimens were analyzed by 10x Genomics based on scRNA-seq [single-cell tagged reverse transcription sequencing (STRT-seq)]. The cells were labeled and differential genes were analyzed by Seurat to complete the statistics and distribution mapping. Then, we used the GO database and KEGG database to analyze the enrichment of divergence genes (**Figure 1A**). We distinguished cell subsets by immune cells’ specific surface markers. Among the 19 subsets in the bubble plot, except for clusters 5 and 18, which are NK cells, the rest are T cells, including CD4 T cells, CD8 T cells, and Treg cells (**Figure 1B**). The percentage of different clusters showed that in AA, cluster 1 and cluster 2 are significantly distinct with the control group (**Figure 1C**).

**Figure 2:**
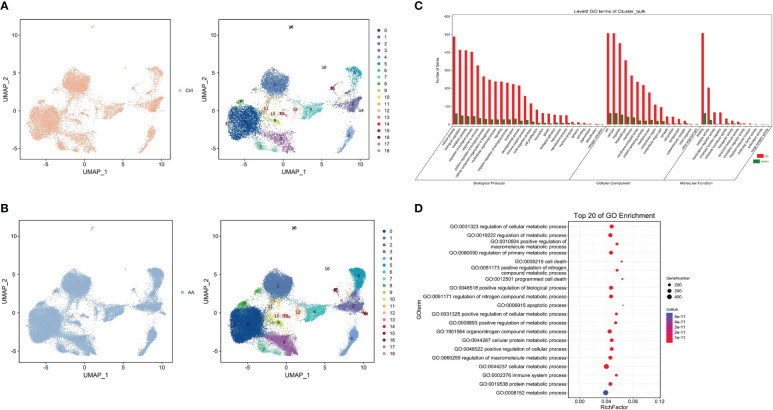
Single cell type identification and enrichment analysis. Uniform manifold approximation and project visualization of the control group **(A)** and aplastic anemia (AA) **(B)** based on single-cell transcriptomes (left). Gene Ontology (GO) enrichment classification histogram depicting the number of up- or downregulated genes in the biological process, cell component, and molecular function ontologies **(C)**. Dot plot representing top 20 enriched GO terms (ranked by Q values) based on bulk RNA-seq **(D)**.

The initial dimension reduction and unsupervised clustering of single-cell transcriptomes classified cells into 19 groups (**Figures 2A, B**). Compared with the control group, the difference of cluster 3 and cluster 5 is rather obvious in AA. At the same time, we conducted GO enrichment analysis from three aspects (biological process, cell component, and molecular function). The research found that, in the biological process part, genes upregulated in the AA group were significantly more than those downregulated (**Figure 2C**). Further study on GO enrichment analysis showed that, compared with the control group, most of the first 20 GO terms in the AA group were related to the regulation of cell metabolism (**Figure 2D**). Therefore, the abnormal regulation of cell metabolism and abnormal expression of related genes may be associated with the occurrence of AA.

### Single-cell mRNA sequencing revealed metabolic differences in aplastic anemia

**Figure 3:**
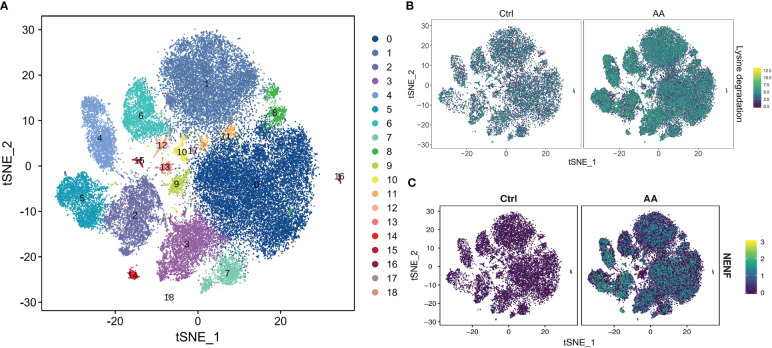
scRNA-seq revealed metabolic differences in AA. t-SNE plot of all cells representing the cell clusters analyzed by scRNA-seq. Each dot represents a single cell; each color corresponds to one cluster **(A)**. t-SNE plot showing the metabolism differences of lysine degradation between AA patients and healthy donors. Colors indicate logarithmic-transformed P values **(B)**. t-SNE plot showing the significantly expressed gene *NENF* in AA patients compared with those in healthy donors **(C)**.

In order to further understand which metabolic processes are unusually regulated and metabolism-related genes are abnormally expressed in patients with AA, we conducted the KEGG pathway and GSEA. So as to display the distribution characteristics of metabolic pathways in different cell subsets, we manufactured the t-distributedstochastic neighbor embedding (t-SNE) map through the R package. The soft k-means clustering algorithm is used to cluster the dimension-reduced data, and cells were clustered into 19 cell types (**Figure 3A**). In the t-SNE difference distribution map of metabolism pathways, it was intuitively seen that lysine degradation was more common in AA (**Figure 3B**). We manufactured the homogenized gene expression into a t-SNE map. The results showed that the expression of the Neustein neurotrophic factor (*NENF*) gene in AA was significantly higher than that in the control group (**Figure 3C**).

**Figure 4:**
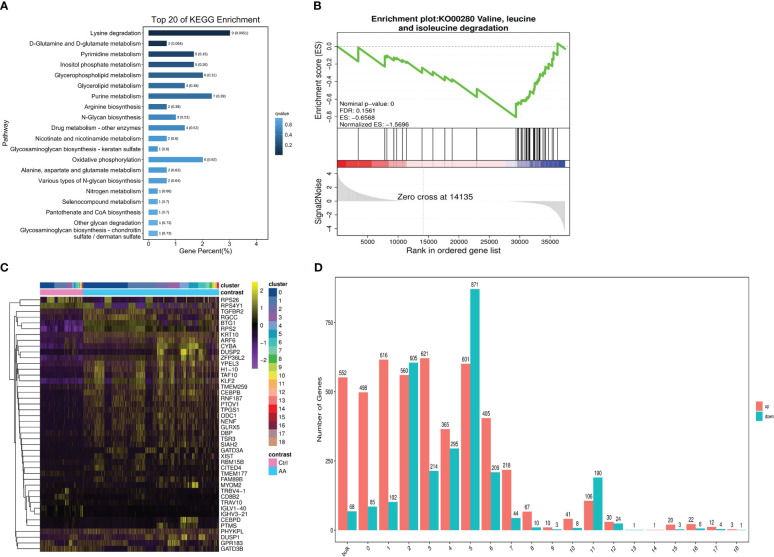
scRNA-seq revealed metabolic differences in AA. Bar chart showing top 20 of the KEGG pathway enriched in cell metabolism compared to those in the control group **(A)**. Gene set enrichment analysis (GSEA) plots of valine, leucine, and isoleucine degradation differences in AA patients compared with those in healthy donors **(B)**. Heat map of differential genetic expression handled with the z-score to normalize gene expression **(C)**. Bar chart showing the number of up- or downregulated genes in bulk and 19 subsets. Red indicates upregulation, and green indicates downregulation **(D)**.

Compared with the healthy donors, analysis found that the dysregulation of lysine degradation in patients with AA had marked statistical significance (AA *vs*. Ctrl, q = 0.0051) (**Figure 4A**). Interestingly, in the GSEA, we found that valine, leucine, and isoleucine degradation–related genes were more abundant in healthy donors (**Figure 4B**), which may indicate that valine, leucine, and isoleucine accumulated multiple times in the AA patients. To sum up, we concluded that the pathobolism of amino acids may play a key important role in the occurrence of AA. Next, we further analyzed from the aspect of metabolizing gene expression. In the heat map, there are significantly more abnormal regulated genes in cluster 3 and cluster 5 in AA compared with the control group (**Figure 4C**). Seurat software was performed to analyze the divisions between the subset of cells. The results were similar to the said GO analysis. The genes upregulated in AA were significantly more than those downregulated. At the same time, the metabolized gene expression in the third and fifth subsets was more obvious than that in other subsets (**Figure 4D**). These findings provided clues for our follow-up research on the mechanism of AA.

### T-lymphocyte metabolism analysis

**Figure 5:**
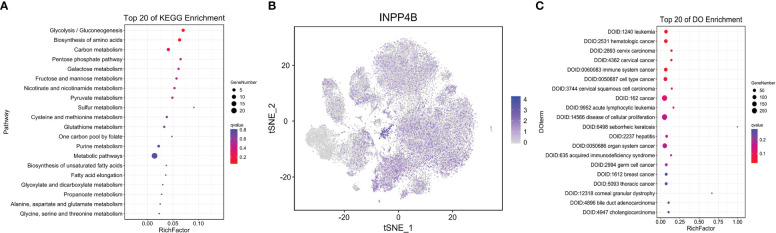
T-lymphocyte metabolism analysis. Dot plot depicting the top 20 KEGG pathway in T lymphocytes. Dot sizes represent the gene number, and dot colors represent Q values **(A)**. t-SNE plot showing the differently expressed gene *INPP4B* in T lymphocytes. All but clusters 5 and 18 are lymphocytes. Each dot represents a single cell. Colors indicate logarithmic-transformed P values **(B)**. Dot plot showing top 20 of disease ontology enrichment in T lymphocytes **(C)**.

As previously mentioned, compared with the control group, the number of abnormally expressed genes in the fifth subset of the AA group is the highest among all subsets. Then, we analyzed the changes of metabolic pathways in the fifth subgroup through the KEGG pathway, listed the metabolic pathways of top 20, and drew dot plots. It was found that the glycolysis/gluconeogenesis metabolic pathways were the most significant (**Figure 5A**). Because most cases of AA are an immune system disorder disease mediated by T cells, this article mainly studies the divergences of immune cell metabolism between the AA group and the control group. Then, we drew a t-SNE map according to the expressed genes through the R package and found that the *INPP4B* gene was specifically expressed in T cells but almost not in NK cells (**Figure 5B**). To check out which diseases may be caused by abnormal metabolic T cells in the process of AA progression, we conducted Disease Ontology analysis, and the results showed that AA was most likely to be converted to leukemia (**Figure 5C**). The said results provide an important basis for our follow-up research on the treatment and prevention of AA.

### Natural killer cell metabolism analysis

**Figure 6:**
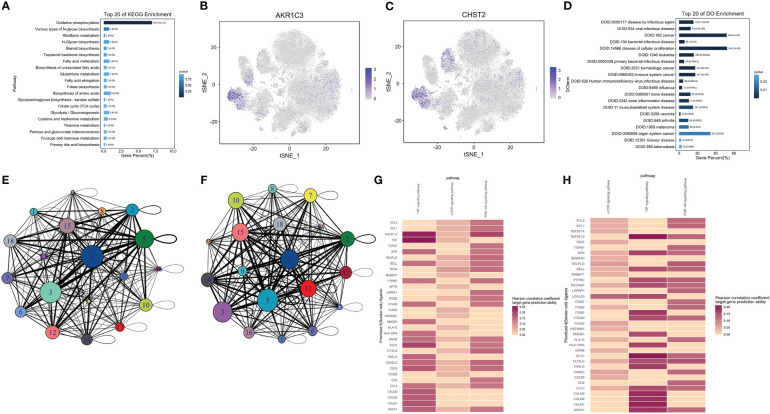
Natural killer (NK) cell metabolism analysis. Bar chart showing top 20 of the KEGG pathway in NK cells. Column length represents the percentage of differential genes, and column colors represent Q values **(A)**. t-SNE plot showing differently expressed genes *AKR1C3* **(B)** and *CHST2* **(C)** in NK cells. Cluster 5 is an NK cell. Each dot represents a single cell; Colors indicate logarithmic transformed P values. Bar chart depicting top 20 of disease ontology enrichment in NK cells **(D)**. Intercellular interaction network between 18 subsets of control **(E)** and AA **(F)**. Molecular interaction states of 36 ligand–receptors between clusters 4 and 5 in the control **(G)** and AA **(H)** groups. Molecules in red indicate that the Pearson correlation coefficient target gene prediction ability is higher.

It was reported that the dysfunction of natural killer (NK) cells may also be related to the occurrence of AA. Then, we analyzed the changes of metabolic pathways in NK cells through the KEGG pathway and found that the oxidative phosphorylation in NK cells in AA was abnormal, which had statistical significance (**Figure 6A**). We continued to explore which metabolism-related genes are abnormally expressed in NK cells and drew the t-SNE map. Interestingly, the expression amount of the *AKR1C3* gene and *CHST2* gene in NK cells is significantly higher than that in other cell subsets (**Figures 6B, C**). *CHST2* (carbohydrate (N-acetylglucosamine-6-O) sulfotransferase 2) gene encodes a sulfotransferase protein, which catalyzes the sulfation of non-reducing n-acetylglucosamine residues and participates in the metabolism of lymphocytes at the inflammatory sites (24). Similar to what was mentioned earlier, we also did the Disease Ontology analysis of NK cells, listed the metabolic pathways of top 20, and drew bar charts. It was found that the metabolic abnormalities of NK cells were the most likely to cause AA to revert to cancer and the disease of cell promotion (**Figure 6D**). The intercellular interaction network between 18 subsets showed that cluster 5 had the strongest correlation among all cell subsets in the AA group (**Figure 6F**), while cluster 4 had the strongest correlation among all cell subsets in the control group (**Figure 6E**), indicating that the communication relationship of subgroup 5 in the AA group is enhanced. Among molecule interactions, the expression of the TNF signaling pathway, mechanistic target of rapamycin (mTOR) signaling pathway, and PI3K-Akt signaling pathway in AA (**Figure 6H**) and control (**Figure 6G**) had the most obvious difference. We further observed that TNFSM12 in TNF signaling was broadly activated in AA, which might contribute to the high reduction of normal blood cells. The metabolic correlation analysis of NK cells in AA provides a new perspective for us to study the mechanism of AA.

## Discussion

T cells were divided into cytotoxic T cells, helper T cells, regulatory T cells, and memory T cells according to their functions and surface markers. After being activated by antigens in the peripheral circulation, naive CD4 T cells proliferate and differentiate into various subsets of T helper cells, including Th1, Th2, and Th17 cells (25). The metabolic reprogramming of T cells enables them to shift from oxidative metabolism to biosynthetic metabolism to support rapid cell growth (26). Activated CD4 T cells require efficient glucose uptake, glycolysis, glutamate decomposition, and lipid synthesis to maintain cell proliferation (27). The enhancement of aerobic glycolysis in cells makes glucose and other nutrients not oxidized in mitochondria to produce ATP but used for the biosynthesis of nucleic acids, lipids, and amino acids (28). The boost of the glycolytic metabolic pathway occurs mostly in activated NK cells (29), T lymphocytes (30), and B lymphocytes (31). Previous studies have shown that pyruvate dehydrogenase is a key enzyme in T-cell glycolysis and oxidative metabolism (32). In our study, we found that the lysine degradation pathway of immune cells in AA is significantly higher than that in normal samples through the single-cell sequencing analysis of peripheral blood clinical samples. Lysine degradation is caused by ϵ-deamination or α-deamination reaction and produces two acetyl coenzyme A and several reductants (33). It has been found that the selective modification of lysine sites in proteins by aminophiles disrupts the interaction between proteins and RNA in the immune response (34). Our results showed that the disturbance of the immune system in AA was also related to the degradation of valine, leucine, and isoleucine. Previous studies indicated that L-amino acid transporters that are composed of Slc7a5 and CD98 induce leucine uptake, activate the mTOR pathway, and affect T-cell metabolism (35). During the activation of T cells and B cells, the transcription of intracellular glutamine transporters SNAT1 and SNAT2 is enhanced (36). It is covered that the NK cell count is decreased and the activity is impaired in patients with Fanconi anemia (37). NK cells mainly rely on oxidative phosphorylation to generate energy and activate downstream to produce interferon-γ (IFN-γ) (38). Oxidative phosphorylation and glycolysis are two major metabolic pathways for energy production and cell function maintenance. In immune cells, oxidative phosphorylation can regulate the formation of memory cells and related inflammatory reactions (39). Several recent studies in the US have shown that Cox10 (a gene encoding the composition of mitochondrial electron transfer chain complex IV) plays an important role in NK-cell antigen-specific amplification and murine cytomegalovirus (MCMV) infection (40).

At the transcriptional level, our research found that the NENF gene was upregulated in the immune cells of AA. Neudesin was initially identified as a secreted protein with neurotrophic activity. It has a conservative cytochrome 5-like heme/steroid binding domain and can activate intracellular signal pathways by binding to G protein–coupled receptors (41). NENF is essential in a variety of biological processes, including neural function, fat metabolism, and tumorigenesis (42). It has been found that neudesin inhibits adipogenesis in mouse embryonic fibroblasts cells 3T3-L1 (3T3-L1) cells through mitogen-activated protein kinase (MAPK) cascade reaction (43). We also detected that *INPP48B* gene was upregulated in the T cells of patients with AA, and *AKR1C3* and *CHST2* were specifically upregulated in NK cells. INPP4B was initially identified as an enzyme that preferentially hydrolyzes the 4-phosphate of phosphatidylinositol-3,4-bisphosphate (PI(3,4)P2), to generate phosphatidylinositol-3-phosphate (PI(3)P) (44). In recent studies, the overexpression of INPP4B in AML cells enhancescolony-forming potential and induces chemotherapy resistance in acute myelocytic leukemia (AML) patients (45). As a soluble enzyme of the aldehyde ketone reductase family, AKR1C3 plays an important role in regulating prostaglandin, the steroid hormone, and retinoic acid metabolism (46). Chst2 encodes a carbohydrate sulfotransferase that catalyzes the sulfation of the C6 position of GlcNAc during keratan sulfate biosynthesis (47).

Through disease ontology enrichment analysis, the abnormal metabolism of T cells is likely to cause AA to develop into leukemia, and the metabolic changes of NK cells are likely to lead to abnormal cell proliferation diseases and tumors. Relevant results have also confirmed that the secondary myelodysplastic syndrome and acute leukemia usually develop from severe AA after immunosuppressive therapy (48). These findings support the broad potential of targeting functional lysine in the human proteome. It has been discovered that drugs acting on the surface receptors of the CTLA-4 and PD-1 can limit the uptake of glucose and amino acids, so as to negatively regulate the activation of T cells (49). Detailed investigations on specific mechanisms of metabolic abnormalities in the immune cells of AA are needed in the future.

## Data availability statement

The data presented in the study are deposited in the CNCB repository, submission of HRA: HRA003544, project: PRJCA012914.

## Ethics statement

The studies involving human participants were reviewed and approved by Shenzhen Science and Technology Innovation Commission. Written informed consent to participate in this study was provided by the participants’ legal guardian/next of kin. Written informed consent was obtained from the individual(s), and minor(s)’ legal guardian/next of kin, for the publication of any potentially identifiable images or data included in this article.

## Author contributions

LH, JH, and YL conceived the project. LH and LW performed the experiments. QZ, JH, and JY analyzed data. QZ wrote the manuscript. All authors contributed to the article and approved the submitted version.
