# Supplementary material for: Single-cell RNA sequencing depicts metabolic changes in children with aplastic anemia
Source: Front Oncol. 2023 Mar 29;13:1075408. doi: 10.3389/fonc.2023.1075408 (PMC10090469; doi:10.3389/fonc.2023.1075408)
Supplement: Supplementary file 1 [file Table_1.docx]

**Figure S1**

| Name | Age | Sex | WBC (10^9/l) | Hb(g/l) | Thrombocytes(10^9/l) |
| --- | --- | --- | --- | --- | --- |
| LJL-AA | 12 | Girl | 3.6 | 53 | 69 |
| LZL-AA | 11 | Boy | 3.3 | 110 | 63 |
| WJL-AA | 11 | Boy | 3.89 | 96 | 55 |
| SLT-AA | 9 | Girl | 3.92 | 113 | 56 |
| XF-AA | 5 | Girl | 3.16 | 73 | 12 |
| CBC-Ctrl | 10 | Boy | 13.3 | 105 | 314 |
| CRC-Ctrl | 1 | Boy | 14.19 | 101 | 361 |
| LJJ-Ctrl | 1 | Boy | 13.09 | 128 | 262 |
